# Human cases of simultaneous echinococcosis and tuberculosis - significance and extent in China

**DOI:** 10.1186/1756-3305-2-53

**Published:** 2009-11-04

**Authors:** Yu Rong Yang, Darren J Gray, Magda K Ellis, Shu Kun Yang, Philip S Craig, Donald P McManus

**Affiliations:** 1 Ningxia Medical University, Yinchuan, Ningxia Hui Autonomous Region, PR China; 2 Molecular Parasitology Laboratory, Queensland Institute of Medical Research, Brisbane, Australia; 3 School of Population Health, University of Queensland, Brisbane, Australia; 4 Teaching Hospital of Ningxia Medical University, Yinchuan, Ningxia Hui Autonomous Region, PR China; 5 Biomedical Sciences Research Institute and School of Environment and Life Sciences, University of Salford, Salford, UK

## Abstract

During analysis of retrospective community survey data, we identified two patients from Xiji County, south Ningxia Hui Autonomous Region with simultaneous echinococcosis and tuberculosis (TB), representing the first such reports for China. As the echinococcosis chronicity increased, the immune profile in both subjects changed from a Th1 to Th2 response, as shown by a TB skin test, originally positive, becoming negative. Such an elevated Th2 immune profile, with subsequent suppression of the Th1 immune response, is a common feature of chronic helminth infections. Given the difficulties in definitive diagnosis, and the potential increased susceptibility for TB infection in patients with advanced echinococcosis, we suggest that combined TB/echinococcosis surveys be undertaken in this area in the future. This would allow early diagnosis of both TB and echinococcosis cases with better prognosis for effective and sustainable treatment outcomes, ultimately reducing associated morbidity and mortality, and also the overall financial costs to the individual and the public health care system in this under developed part of China.

## Findings

Tuberculosis (TB) affects over five million people in China, 80 percent living in rural areas [1]. Community surveys in 1988-89 in rural townships of Xiji County in south Ningxia Hui Autonomous Region (NHAR), northwest China indicated a TB prevalence ranging from 0.6-12.4% (mean 2.2%) [2]. Helminthiases are also extremely common in China [3]. In Xiji, the prevalence of echinococcosis ranges from 0-7.4% (mean 2%) and from 1-8.1% (mean 2.5%) for the cystic (CE) and alveolar AE forms, respectively [4]. Given the similarities in their morbidities, specific diagnosis of TB and echinococcosis in co-infected individuals is difficult which may account for the limited number of reports of simultaneous infections globally [5].

Retrospective community survey data for TB and echinococcosis [2,4] revealed co-endemic TB and echinococcosis in a number of townships within Xiji County. The range of TB prevalence (0.6-12.4%) in the TB/echinococcosis co-endemic townships was similar to that of the entire county [2] but the mean prevalence (3.9% v 2.2%) was significantly higher (Chi-square; P = 0.036). Prevalence of echinococcosis in the townships ranged from 0.8%-9% (mean 4.5%). It is noteworthy that while the surveys were up to 15 years apart, the incubation period for AE/CE is 10-15 years [6] and so primary *Mycobacterium tuberculosis *and *Echinococcus *infections would have been occurring at the same time.

We describe two survey subjects with simultaneous echinococcosis and TB, representing the first such reports for China. Both subjects indicated by questionnaire having received Bacillus Calmette-Guerrin (BCG) vaccination during childhood, confirmed by a characteristic raised skin vaccination scar. Approval for the surveys was given by the Ethics Committee of Ningxia Medical College; written consent to participate was obtained from both patients.

The first, an 18-year-old male reported to Xiji County Hospital in 2006 with malaise, weakness, fatigue and a minor cough. Ultrasound (US) and Computed Tomography (CT) scans identified an uneven hypo-echo image with two irregular lesions in the right liver suggestive of P3 stage AE [6] (Fig. 1.1a). Chest x-ray images showed a number of small shadows scattered on both sides of his lungs, the largest being in the lower lobe of the left lung; CT scans showed a defined edge (Fig. 1.1c &1.1d), possibly a CE lesion. Further US and serology (highly positive for anti-Em18 antibodies [7]) confirmed P3 stage AE in the liver with possible metastasis to the lungs. He was prescribed albendazole (ABZ) (10 mg/kg/day) and advised to attend the annual clinical follow-up thereafter.

**Figure 1:**
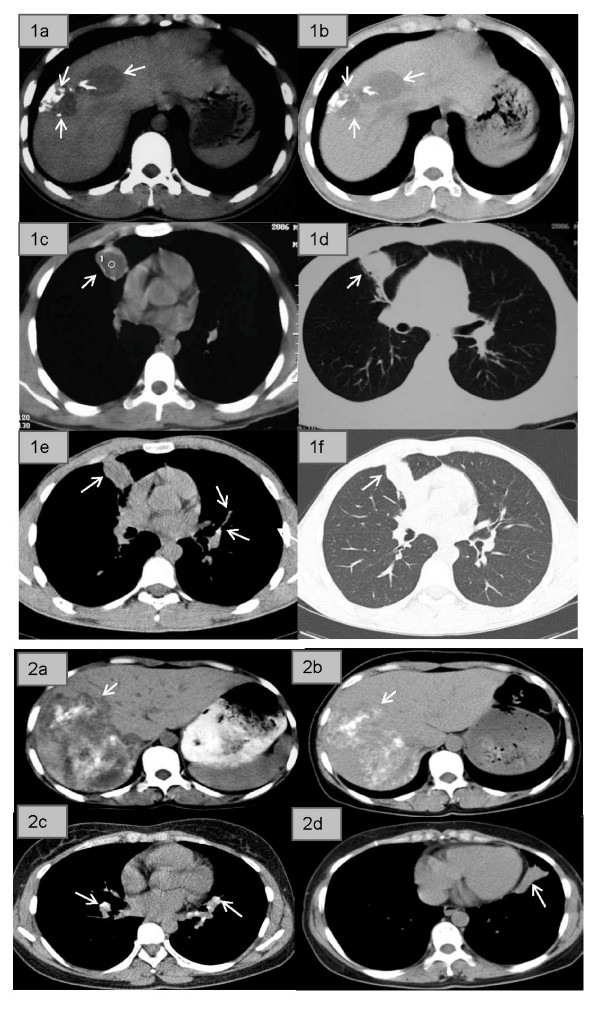
**CT scans of patient 1 (18-year-old male) (panels 1a-f) and patient 2 (36 year-old female) (panels 2a-d)**. Panel 1a shows the liver in 2006 showing two irregular lesions located in the right lobe with scattered calcifications. Panel 1b is a follow-up scan of the liver taken in 2008 showing that the lesions had shrunk in size after treatment with albendazole. Panels 1c and 1d show the lung in 2006 having a lesion with the density of liquid, a clear edge, and surrounding inflammatory infiltrate extending to the anterior pleura. Panels 1e and 1f are follow-up lung CT scans taken in 2008 showing the lesion had shrunk in size. Panel 2a shows the liver in 2003 with a large irregular lesion located in the right lobe having scattered calcifications and liquefactions within it. Panel 2b shows the liver in 2008 indicating that the same lesion had not changed in size but with increased calcification and decreased liquefaction areas present. Panels 2c and 2d show the lung in 2008. Panel 2c shows a number of calcified lymph nodes on both sides of the upper and middle lung hila (indicating previously active TB). Panel 2d shows scar tissue (approximately 1.5 × 5 cm) in the right lower lobe of the lung without related lymph node calcification.

The patient had been previously diagnosed with pulmonary TB by chest x-ray in 2000; he had a positive tuberculin skin test and acid fast bacilli were present in his sputum. Clearly, the earlier BCG vaccination did not protect. A routine clinical examination in 2003 indicated that the TB had been cured following three years of regular TB chemotherapy [8]. X-rays taken in 2006 during the Xiji County Hospital visit were inconclusive and further tests to assess TB status were undertaken. A negative tuberculin skin test and sputum test ruled out relapsed TB. At follow-up in 2008, the tuberculin skin test had reverted, becoming positive. CT scans of the liver showed that the AE lesion had shrunk markedly in size (Fig. 1.1b) indicating effective ABZ treatment. Serology was marginally positive for anti-Em18 antibodies. A CT scan of the chest showed a fibre-like scar in the middle right lung and a calcified lymph node in the middle right hilum (Fig. 1.1e). Moreover, the lesion was irregular, with greater density (Fig. 1.1e and 1.1f) compared with the 2006 CT scan (Fig. 1.1c and 1.1d). The clinical diagnosis was confirmed CE in the lung and effective ABZ treatment.

The second subject was a 36 year-old female referred to Ningxia Teaching Hospital, Yinchuan city in 2003 complaining of constant abdominal pain. Her BCG vaccination had not been protective as chest X-rays in 1998 showed TB lesions on both lungs and she had a positive tuberculin skin test but negative TB sputum test. Despite having been prescribed TB drugs [8], that she had taken intermittently, there were concerns that the TB had invaded her abdomen. X-rays of the chest and abdomen were normal and the tuberculin skin and sputum tests were negative. Abdominal CT (Fig. 1.2a) indicated an advanced stage (P4) liver AE lesion with vascular and biliary involvement of the right liver, with scattered calcifications within the lesion. Serology [7] for anti-EgB, but not anti-Em18, antibodies, was positive. She was prescribed ABZ and followed-up annually by US and questionnaire examination [4].

In 2005, questionnaire analysis revealed she had two recent episodes of severe coughing with mucus and white gelatinous membranous materials expelled, the first in late 2004 following 18 months of ABZ treatment and the second in the spring of 2005. Microscopic examination after the second episode showed the presence of CE-laminated-membranes [9]. The patient was thus diagnosed with simultaneous AE and CE. Continuous ABZ was prescribed with annual follow-up to monitor treatment efficacy.

Chest CT scans in 2008 showed scars on the top and middle lobes of both sides of the lung, calcification on both sides of their related hilar lymph nodes (Fig. 1.2c), and a 5 × 1.5 cm density tissue scar in the lower right lobe (Fig. 1.2d), indicating ABZ had affected the CE lesion. A liver CT scan showed the same AE lesion (Fig. 1.2b) recorded in 2003 (Fig. 1.2a), with marginally fewer liquefaction areas than before, indicating ABZ had had limited effect. Em18 serology and the tuberculin skin and sputum tests for TB were negative.

These are the first cases of simultaneous TB and echinococcosis reported in China; both patients presented with simultaneous AE and CE as well, a feature not uncommon for NHAR [10]. The significantly increased TB prevalence in residents of Xiji townships co-endemic for TB and echinococcosis compared with those from townships endemic only for TB, suggested the increase may be due to co-infection with *Echinococcus*.

As the echinococcosis chronicity increased in the two patients (following the latency period after ingestion of *Echinococcus *eggs), the immune profile appeared to change from a Th1 to Th2 response, reflected by the positive tuberculin skin test becoming negative. This corresponded mainly with the progression of AE from early (P1 & P2) to advanced (P3 & P4) stage disease [6], as the infiltrative growth of an AE lesion releases considerably more parasite antigen constantly stimulating the host immune system [6]. An elevated Th2 immune profile and subsequent suppression of the Th1 immune response is common in helminth infections [11]. With a suppressed Th1 immune profile the hosts' ability to detect and respond to viruses, bacteria and other pathogens is impaired [12] and Th1-based vaccines can become ineffective.

Despite BCG vaccination, both subjects developed TB and were skin-test-positive. Following TB treatment, they developed non-specific symptoms resembling a relapsed TB infection; however both cases then became skin test-negative and were subsequently diagnosed with advanced stage (P3 or P4) AE. Given the length of the incubation period of an *Echinococcus *infection, it is most likely that the cases were infected prior to contracting TB.

Albendazole can vary in its efficacy against echinococcosis, particularly AE [6], exemplified by these two patients. In the first, treatment was effective as shown in 2008 by CT scans of the liver showing regression of the AE lesion, with his immune profile reverting to Th1 as shown by the tuberculin skin test reverting to positive. In contrast, treatment of the female subject was largely ineffectual; the large AE lesion showed no reduction in size and the tuberculin test remained negative.

Similarities in the symptoms of TB and echinococcosis and the immune progression of both diseases make their definitive diagnosis difficult. A TB infection can often mask the symptoms of early stage echinococcosis, particularly pulmonary echinococcosis. Suppression of the immune system can then promote the progression to later stage echinococcosis or secondary metastases in the case of AE. Moreover, subjects with advanced stage echinococcosis have a pronounced Th2 immune profile [11], mirrored in both study subjects by their negative tuberculin skin test. A previous report of helminth infected Ethiopian immigrants showed they responded poorly to skin testing for TB compared with immigrants who had been de-wormed [13] indicating that this immune modulation may compromise the diagnostic efficacy of the tuberculin skin test. Further, studies have also shown that severe AE or CE cases exhibit poor lymphocyte responses both in vivo and in vitro with the Th1 immune response being strongly suppressed in patients with advanced disease [12,14].

A recent study has suggested a mechanism whereby concomitant systemic helminth infections predispose to the development of active tuberculosis in humans [15]. By analysing the cellular responses to mycobacterial antigens in patients who had latent tuberculosis with or without filarial infection, the study demonstrated that filarial infection coincident with *M. tuberculosis *infection significantly diminishes *M. tuberculosis*-specific Th1 (interleukin [IL]-12 and IFN-gamma) and type 17 T helper (Th17; IL-23 and IL-17) responses related to increased expression of cytotoxic T lymphocyte antigen (CTLA)-4 and programmed death (PD)-1. These findings reinforce our earlier comments regarding vaccine use in helminth - endemic countries in that those requiring a Th1 or Th17 response for efficacy may not function optimally in the presence of a chronic helminth coinfection.

Xiji County is underdeveloped and poor and the local hospitals generally lack adequate facilities and technical expertise, with laboratory diagnosis often unhelpful. The majority of TB cases are usually diagnosed by clinical features, exposure history, a positive tuberculin test and the presence of bacteria in sputum by microscopy. This latter method is rapid, but it is relatively insensitive if insufficient sputum is provided, or if few bacteria are present [16]. Given there is co-endemic TB and echinococcosis in Xiji, the difficulties in definitive diagnosis, and the potential increased susceptibility for TB infection in patients with advanced echinococcosis, we suggest that combined TB/echinococcosis surveys be undertaken in this area in the future. This would allow early diagnosis of both TB and echinococcosis cases with better prognosis for effective and sustainable treatment outcomes, ultimately reducing associated morbidity and mortality, and also the overall financial costs to the individual and the public health care system in this under developed part of China.

## Competing interests

The authors declare that they have no competing interests.

## Authors' contributions

YRY, PSC and DPM conceived the study, YRY carried out the survey and questionnaire work, SKY undertook the US and CT scans, YRY, DG, ME and DPM analysed the data, and YRY, DG, ME and DPM wrote the manuscript.
